# Long-Term Dynamic Humoral Response to SARS-CoV-2 mRNA Vaccines in Patients on Peritoneal Dialysis

**DOI:** 10.3390/vaccines10101738

**Published:** 2022-10-18

**Authors:** Borja Quiroga, María José Soler, Alberto Ortiz, Ron T. Gansevoort, Alba Leyva, José Rojas, Patricia de Sequera

**Affiliations:** 1 Nephrology Department, Hospital Universitario de la Princesa, 28006 Madrid, Spain; 2 Nephrology Department, Vall d’Hebrón University Hospital, 08035 Barcelona, Spain; 3 IIS-Fundación Jimenez Diaz, School of Medicine, Universidad Autónoma de Madrid, Fundación Renal Iñigo Alvarez de Toledo-IRSIN, REDinREN, Instituto de Investigación Carlos III, 28040 Madrid, Spain; 4 Dept. Internal Medicine, University Medical Center Groningen, University of Groningen, Postbus 30001, 9700 RB Groningen, The Netherlands; 5 R&D Department, VIRCELL SL, 18001 Granada, Spain; 6 Nephrology Department, Infanta Leonor University Hospital, 28031 Madrid, Spain

**Keywords:** SARS-CoV-2, COVID-19, humoral response, anti-spike antibodies, peritoneal dialysis, chronic kidney disease, booster

## Abstract

Introduction. Patients on peritoneal dialysis (PD) present an impaired humoral response against SARS-CoV-2, at least after the initial vaccination and booster dose. Until now, the effect of a fourth dose has not been established. The aim of the present study is to evaluate the long-term dynamics of the humoral response of PD patients to multiple doses of SARS-CoV-2 vaccines, focusing on the effect of the fourth dose. Methods. This is an analysis of the prospective and multicentric SENCOVAC study. We included patients on PD without additional immunosuppression that had received at least 3 SARS-CoV-2 mRNA vaccine doses. We evaluated anti-spike antibody titers after the initial vaccination, third and fourth doses, using prespecified fixed assessments (i.e., baseline, 28 days, 3, 6, and 12 months after completing the initial vaccine schedule). Breakthrough infections were also collected. Results. We included 164 patients on PD (69% males, 62 ± 13 years old). In patients who had received only two doses, the rates of positive humoral response progressively decreased from 96% at 28 days to 80% at 6 months, as did with anti-spike antibody titers. At 6 months, 102 (62%) patients had received the third vaccine dose. Patients with the third dose had higher rates of positive humoral response (*p* = 0.01) and higher anti-spike antibody titers (*p* < 0.001) at 6 months than those with only 2 doses. At 12 months, the whole cohort had received 3 vaccine doses, and 44 (27%) patients had an additional fourth dose. The fourth dose was not associated to higher rates of positive humoral response (100 vs. 97%, *p* = 0.466) or to statistically significant differences in anti-spike antibody titers as compared to three doses (*p* = 0.371) at 12 months. Prior antibody titers were the only predictor for subsequent higher anti-spike antibody titer (B 0.53 [95%CI 0.27–0.78], *p* < 0.001). The 2 (1.2%) patients that developed COVID-19 during follow-up had mild disease. Conclusions. PD presents an acceptable humoral response with three doses of SARS-CoV-2 vaccines that improve the progressive loss of anti-spike antibody titers following two vaccine doses.

## 1. Introduction

Patients with chronic kidney disease (CKD) are at high-risk for complications due to coronavirus disease-19 (COVID-19) [1]. Among the CKD spectrum, kidney transplant recipients and dialysis patients present inherent characteristics that make them more vulnerable to severe acute respiratory syndrome coronavirus 2 (SARS-CoV-2) infection and condition a worse prognosis [2,3]. Peritoneal dialysis (PD) is a home dialysis therapy that has been one of the preferred techniques for patients requiring renal replacement therapy (RRT), especially for incident patients, during COVID-19 pandemic [4]. In contrast, patients on in-center hemodialysis have presented higher rates of COVID-19 as they are required to attend a healthcare center 3 to 6 times per week and to use healthcare transportation; which may be shared, both factors potentially linked to SARS-CoV-2 transmission [5]. Beyond the first waves of the pandemic, in which mortality rates were high, vaccination and new less lethal variants have changed the paradigm and prognosis of COVID-19 [6,7,8,9,10].

In patients on PD, SARS-CoV-2 vaccination has demonstrated to be safe and to promote early and strong seroconversion at least with the initial schedule (i.e., two doses of mRNA-based vaccines [BNT162b2, mRNA-1273] or ChAdOx1-S or one dose of Ad26.COV.2) [6,11]. However, anti-spike antibody titers in PD decrease over time, as is the case for other RRT patients, potentially leading to suboptimal protection against SARS-CoV-2 [12,13]. In this regard, a booster (or third) dose was considered early for these vulnerable patients. Although evidence on the effectivity of the third dose on patients in PD is scarce, some studies have suggested a robust humoral response rise [14,15]. More importantly, a booster dose has been demonstrated to seroconvert patients that had previously lost their humoral immunity against SARS-CoV-2 [16,17]. Some countries have provided a fourth vaccine dose to some groups of vulnerable or elderly patients. However, we are not aware of assessments of the effects of the fourth dose in patients on PD.

The aim of the present study is to evaluate the long-term humoral response to SARS-CoV-2 mRNA vaccines in patients on PD, focusing on the anti-spike antibody titers after the third and fourth doses.

## 2. Materials and Methods

### 2.1. Design

This is an analysis of the SENOVAC study. SENCOVAC is a multicentric, observational and prospective study that aimed to evaluate the safety and humoral response of SARS-CoV-2 vaccines across the CKD spectrum (kidney transplant recipients, patients on hemodialysis, PD and with advanced CKD). In Spain, the choice of the specific type of vaccines in the initial schedule (i.e., BNT162b2 [Pfizer-BioNTech^®^], mRNA-1273 [Moderna^®^], ChAdOx1-S [AstraZeneca^®^] or Ad26.COV.2 [Janssen^®^]) was made by the national health authority and not by SENCOVAC investigators. In addition, mRNA-based vaccines (i.e., BNT162b2 [Pfizer-BioNTech^®^], mRNA-1273 [Moderna^®^]) were used for the third and fourth doses, and again, the specific vaccine used was determined by the Spanish health authority.

### 2.2. Population

For the present analysis, we included patients on PD that had received at least 3 doses of mRNA-based vaccines. During the follow-up, the humoral response (anti-SARS-Cov-2 spike antibodies) was determined at baseline (before vaccination), 28 days, 3 months, 6 months, and 12 months after completing the initial vaccination schedule of 2 mRNA vaccine doses. We excluded patients with solid organ transplantation, active neoplasia, primary immunodeficiencies, human immunodeficiency virus, and patients who had received immunosuppressive treatment within 6 months before vaccination. Between the 6-month and 12-month assessments, some patients received a fourth dose of vaccine, following health authority policy that was independent of SENCOVAC researchers. For the present analysis, we divided the cohort into two groups depending on the vaccination status (three or four doses) at 12 months. This permitted us to compare humoral responses in patients on PD with three or four doses of SARS-CoV-2 vaccines. In addition, at month 6, some patients had received the third (or booster) dose, allowing us to compare patients with only the initial complete vaccination to patients with the initial complete vaccination plus the third dose at this time point.

### 2.3. Objectives and Outcomes

The primary objective was to evaluate the humoral response during the 1-year follow-up after SARS-Cov-2 vaccination in patients on PD and the factors associated with the humoral response. Secondary objectives included the analysis of the impact of third and fourth doses on anti-spike antibody titers and the registration of breakthrough COVID-19.

### 2.4. Variables

At baseline, epidemiological (age and sex) data, etiology of CKD, and dialysis modality were registered. At baseline, 28 days, 3, 6, and 12 months after completing the initial vaccination, serum samples were obtained and sent to a central laboratory where anti-spike antibodies were measured. Anti-spike antibodies were tested by a CE-marked quantitative chemiluminescence immunoassay (CLIA, COVID-19 Spike Quantitative Virclia^®^ IgG Monotest, Vircell SL, Granada, Spain), with a sensitivity and specificity of 96% and 100%, respectively, that detects IgG antibodies against the SARS-CoV-2 spike protein. This assay was calibrated against the First WHO International Standard for anti-SARS-CoV-2 human immunoglobulin (NIBSC code: 20/136), and results were expressed as IU/mL. According to performance studies by the manufacturer, titers ≤32 IU/mL were considered negative, between 32 and 36 equivocal and >36 IU/mL positive, reflecting the presence of anti-spike IgG antibodies resulting from previous infection or vaccination. The highest titer that was measurable was 10,000 UI/mL. Thus, a titer of 10,000 UI/mL means 10,000 UI/mL or higher. During the 1-year follow-up, breakthrough SARS-CoV-2 infections were registered. The definition of infection required a positive rt-PCR or antigen test.

### 2.5. Statistics

Data is expressed as median (interquartile range) or percentage depending on the type of variables. We used the Fisher test and Mann–Whitney test for comparing categorical and continuous variables, respectively. Linear regression analysis was performed to assess variables associated to 12-month anti-spike antibody titers in adjusted models, including confounders. SPSS version 26.0 (IBM Corp., Armonk, NY, USA) was used for statistics and GraphPad Prism version 9.02 (GraphPad Holdings, LLC, CA 92037, USA) for plotting.

## 3. Results

### 3.1. Baseline Characteristics

We included 164 patients on PD (69% males, 62 ± 13 years old). Eighty-five patients (52%) were on continuous ambulatory peritoneal dialysis (CAPD) and 79 (48%) on automated peritoneal dialysis. Regarding the etiology of CKD, 44 (27%) had a glomerular disease, 31 (19%) diabetic kidney disease, 28 (17%) nephroangiosclerosis, 24 (15%) unknown disease, 18 (11%) autosomal polycystic kidney disease, 10 (6%) interstitial nephritis, and 9 (5%) others (Supplementary Table S1).

### 3.2. Humoral Response after the Initial Vaccination Schedule

Initial vaccination was performed using mRNA-based vaccines, 123 (75%) patients received mRNA-1273, and 41 (25%) received BNT162b2.

**Figure 1:**
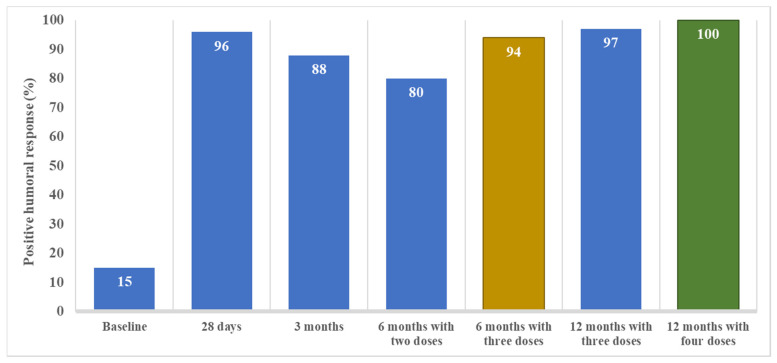
The positive humoral response over time from baseline (prior to SARS-CoV-2 vaccination) to 12 months after completing the initial vaccine schedule (i.e., 2 doses of mRNA vaccine) in CKD patients on peritoneal dialysis. A positive humoral response was defined as anti-spike antibody titers above 36 UI/mL. Note that samples obtained at 6 and at 12 months following the initial vaccine schedule of two doses of mRNA-based vaccines are divided into patients that had received 2 (just the initial schedule) or 3 doses (initial schedule plus one booster dose) at the 6-month timepoint or 3 or 4 doses (12-month timepoint).

**Figure 2:**
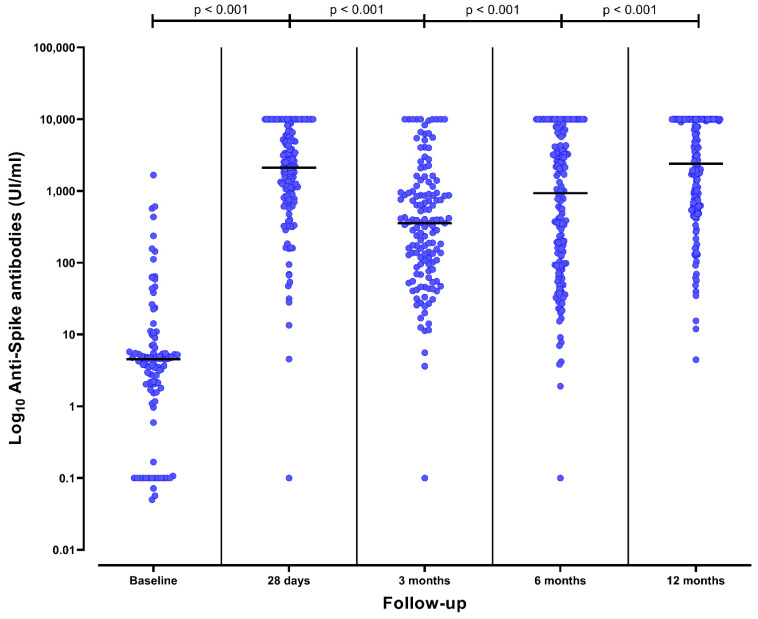
Anti-spike antibody titers over time from baseline (prior to SARS-CoV-2 vaccination) to 12 months after completing the initial vaccine schedule (i.e., 2 doses of mRNA vaccine) in CKD patients on peritoneal dialysis. The black line represents the median value of each strata.

Humoral response varied during the follow up. At baseline, 15 (15%) of the 102 tested patients presented positive humoral responses because of previous SARS-CoV-2 infection. Twenty-eight days after complete vaccination, 138 (96%) of the 143 tested patients presented positive humoral responses (Figure 1). Among the 81 patients without a humoral response at baseline, 78 (96%) seroconverted after vaccination. Anti-spike antibodies were significantly higher one month after vaccination than at baseline (4 [2–7] UI/mL vs. 2109 [775–5982] UI/mL, *p* < 0.001) (Figure 2).

Three months after vaccination, 123 (88%) of the 139 tested patients presented a positive humoral response (Figure 1). Among the 123 patients with previous positive humoral response, 10 (8%) had a negative humoral response at 3 months and one (1%) had an equivocal response. Anti-spike antibodies significantly decreased at 3 months in comparison to one-month titers (356 [95–992] UI/mL vs. 2109 [775–5982] UI/mL, *p* < 0.001) (Figure 2).

In the absence of a booster (third dose), a positive humoral response was present at 6 months in 43 (80%) of the 54 patients who at this timepoint had only received the initial two vaccine doses (*p* = 0.040 vs. 3 months). The anti-spike antibodies significantly decreased at 6 months in comparison to 3-month titers (100 [37–365] UI/mL vs. 356 [95–992] UI/mL, *p* < 0.001). During the initial 6-months of follow-up, patients with the absence of a booster presented a decreased in anti-spike antibodies (*p* for trend < 0.001).

### 3.3. Humoral Response after the Third Dose at 6 Months

Among the 164 patients that received the third dose, 133 (81%) received mRNA-1273 and 31 (19%) BNT162b2.

At 6 months, 139 (89%) of the 157 tested patients presented a positive humoral response (Figure 1). At this time point, 102 (62%) patients had received the third dose. Positive humoral response at 6 months was achieved more frequently in patients that had received the third dose than in a dose with only two doses (96/102 vs. 43/54, *p* = 0.01). Sixteen patients (10%) and 2 patients (1%) presented a negative and uncertain humoral response, respectively, at 6 months. Among negative patients, 12/16 (75%) had not received the third vaccine dose before the 6-month assessment. In contrast, the two patients with uncertain humoral response had received the third dose.

**Figure 3:**
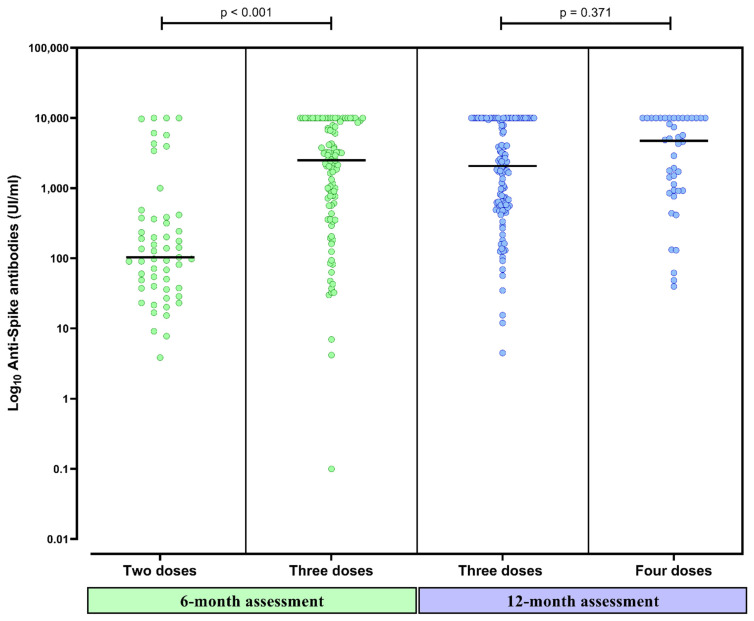
Anti-spike antibody titers 6 months after completing the initial vaccine schedule in CKD patients on peritoneal dialysis who had received two and three doses of SARS-CoV-2 vaccines and titers 12 months after completing the initial vaccine schedule in patients with three and four doses. The black line represents the median value of each strata.

Anti-spike antibody titers significantly increased at 6 months in comparison to 3 months (931 [96–6035] UI/mL vs. 356 [95–992] UI/mL, *p* < 0.001) (Figure 2). Patients that had received a third dose had higher anti-spike antibody titers at 6 months than patients with only two doses (2499 [564–8657] UI/mL vs. 100 [37–365] UI/mL, *p* < 0.001) (Figure 3). The median time elapsed between the third dose and the 6-month assessment was 32 (20–51) days.

### 3.4. Humoral Response after the Fourth Dose at 6 Months

Forty-four patients (27%) had received a fourth dose (31 [70%] mRNA-1273 and 23 [30%] BNT162b2). At 12 months, 160 (98%) of the 164 tested patients presented a positive humoral response (Figure 1). Having received the fourth dose did not significantly increase the already high rate of positive humoral response (44/44 vs. 114/118, *p* = 0.466). Three patients presented a negative humoral response and one presented an uncertain response. None of them had received the fourth dose. Anti-spike antibodies significantly increased at 12 months in comparison to 6-month titers (2391 [614–10,000] UI/mL vs. 931 [96–6035] UI/mL, *p* < 0.001) (Figure 2). Patients that had received the fourth dose had higher anti-spike antibody titers in comparison to those having received three doses, although these differences were not statistically significative (4933 [919–10,000] UI/mL vs. 2065 [560–10,000] UI/mL, *p* = 0.371) (Figure 3). The median time elapsed between the fourth dose and the 12-month assessment was 64 (27–80) days.

### 3.5. Factors Associated with Stronger Humoral Response at 12 Months

In a model adjusted for age, sex, type of mRNA vaccine, and having received the fourth dose, anti-spike antibody titers at 6 months were the only independent factor associated with a stronger humoral response at 12 months (Table 1). There was a lack of association between anti-spike antibody titers and the etiology of CKD, PD technique, or treatment with renin–angiotensin–aldosterone system inhibitors and erythropoietic stimulant agents.

### 3.6. Breakthrough Infections

Only two patients (1.2%) presented a SARS-CoV-2 infection during follow-up. The clinical presentation was mild. One patient had not received the third vaccine dose at the time of COVID-19 and the other developed COVID-19 before the fourth dose (Table 2). Both had last known anti-spike antibody titers below 5000 UI/mL.

## 4. Discussion

The key finding of this analysis of the SENCOVAC study is that patients on PD achieve high rates of positive anti-spike humoral response after the third dose of mRNA-based SARS-CoV-2 vaccines that reverse the progressive decrease of anti-spike antibodies over time observed following two doses. A fourth dose may further increase the humoral response in some patients and result in higher rates of patients with high antibody titers. Patients on PD belong to the especially vulnerable group of kidney failure patients; thus the objective of vaccination against SARS-CoV-2 should include not only achieving a positive humoral response but also high and long-lasting anti-spike antibody titers, as this has been related to lower rates of breakthrough infections and better prognosis [18].

Although there is scarce information on the effectivity of a fourth SARS-CoV-2 vaccine dose in patients on PD, real-world data, including hemodialysis patients, shows a positive effect on the humoral response, even against the Omicron variant [19]. In this regard, repetitive immunological hits (i.e., breakthrough infections and multiple vaccine doses) improve protection against the most recent SARS-CoV-2 variants that now predominate [20]. From the epidemiological point of view, it is prudent to estimate the lower efficacy of first-generation vaccines against novel and future SARS-CoV-2 variants that may more negatively impact vulnerable patients such as CKD patients on RRT [21]. To address this enhanced risk of infection and complications, a multi-vaccine strategy may reduce the rates of suboptimal responses, especially in immunosuppressed patients [21]. However, a recent study of a hemodialysis cohort showed that booster doses were beneficial in virus-naive patients but not in SARS-CoV-2–recovered patients, at least in terms of cellular immunity [22]. Therefore, efforts should be directed at maintaining an optimal immune response, especially in patients who have not been infected or with lower antibody titers, as neutralizing antibody titers are related to higher protection [18]. In this regard, there is insufficient information regarding the optimal cut-off point for anti-spike antibody titers that provide optimal protection against COVID-19 and against severe complicated COVID-19. In our study, only two infections were detected, and both were mild and did not need hospital admission. Both the immune response to vaccines and the successful limitation of exposure to the virus may have contributed to this observation [23].

The progressive decrease in antibody titers over time after each vaccination dose is a cause for concern. Previous reports have shown that 3 to 6 months after initial vaccination and booster doses, significant anti-spike titer decline is observed in dialysis patients [12,24]. At present, individualizing additional dose prescriptions is probably the best strategy to obtain an optimal immune response, avoiding the risk of hyperstimulation and immune exhaustion [25,26]. Vulnerable patients with lower antibody titers or negative humoral response are the highest risk group. Some factors have been proposed to predict an early decline in antibody titers or a lower immediate humoral response. In agreement with our data, the previous humoral response is probably the better predictor of response to successive booster vaccine doses. Additionally, factors such as immunosuppressive drugs or conditions, obesity, older age, absence of previous or breakthrough COVID-19 or lower vaccine dose (number of doses or type of vaccine) can predict a suboptimal immunological response in CKD patients on RRT [6,8,9,10,27]. Thus, these factors should be considered when assessing the risks of not receiving early additional booster vaccine doses.

Our study presents some limitations to be acknowledged. First, the inherent bias of observational studies. However, performing a randomized clinical trial with a placebo group is ethically unacceptable. In addition, the evidence provided by our study can be extrapolated to clinical practice as it is based on real-world data, and both the timing and type of vaccines were determined by a stakeholder external to the study, the health authorities. Second, the low rate of breakthrough infections does not allow an assessment of the efficiency of SARS-CoV-2 vaccines in PD. This is probably due to the sample size but also due to the dynamic changes of the pandemic (new variants, vaccine protocols) that have attenuated its effects. Indeed, some asymptomatic cases can go unnoticed, as periodic SARS-CoV-2 screening was not performed in ambulatory patients such as those on PD. This may have underestimated the infection rate, but it is unlikely to have missed severe COVID-19. Third, the observational design of the study may have resulted in missing data.

In conclusion, patients on PD present an acceptable humoral response with three doses of SARS-CoV-2 vaccines that improve on the natural history of progressive loss of anti-spike antibody titers following two vaccine doses. Adding a fourth vaccine dose did not significantly improve the already high positive humoral response rate. However, there was some suggestion of a stronger immune response in terms of anti-spike antibody titers, and all patients that received the fourth dose developed a positive humoral response. Thus, assessment of anti-spike antibody titers may identify vulnerable patients, such as PD patients, that may derive the most benefit from further booster vaccine doses, leading to individualized booster prescriptions according to immunological background and risk for complications.

## Figures and Tables

**Table 1:** Multivariate analysis using adjusted linear regression for factors associated with higher anti-spike antibody titers at 12 months.

	B (95%CI)	*p*
Age (years)	44 (−52, 141)	0.913
Gender (male)	1726 (−519, 3972)	0.129
mRNA-based vaccine (mRNA-1273)	−914 (−3054, 1225)	0.394
Fourth dose (yes)	2086 (−386, 4560)	0.096
Anti-Spike antibody titers at 6 months (per UI/mL)	0.530 (0.274, 0.786)	<0.001

**Table 2:** Characteristics of patients that developed COVID-19 during follow-up.

Patient	Timing of COVID-19	Initial SARS-CoV-2 Vaccine	Third SARS-CoV-2 Vaccine Dose	Last Anti-Spike Titers Prior to COVID-19 (mUI/L)	Infection Severity	Anti-Spike Titers Post-COVID-19 (mUI/L)
1	Before 3rd dose	BNT162b2	--	1065	Mild symptoms without admission	6599
2	Before 4th dose	mRNA-1273	mRNA-1273	3135	Mild symptoms without admission	10,000

## Data Availability

Data available on request due to restrictions.

## Supplementary Materials

The following are available online at https://www.mdpi.com/article/10.3390/vaccines10101738/s1, Table S1: Baseline characteristics of the participants included in each humoral response assessment.

## References

[1] Williamson E.J., Walker A.J., Bhaskaran K., Bacon S., Bates C., Morton C.E., Curtis H.J., Mehrkar A., Evans D., Inglesby P. (2020). Factors Associated with COVID-19-Related Death Using OpenSAFELY. Nature.

[2] Goffin E., Candellier A., Vart P., Noordzij M., Arnol M., Covic A., Lentini P., Malik S., Reichert L.J., Sever M.S. (2021). COVID-19-Related Mortality in Kidney Transplant and Haemodialysis Patients: A Comparative, Prospective Registry-Based Study. Nephrol. Dial. Transplant..

[3] Hilbrands L.B., Duivenvoorden R., Vart P., Franssen C.F.M., Hemmelder M.H., Jager K.J., Kieneker L.M., Noordzij M., Pena M.J., de Vries H. (2020). COVID-19-Related Mortality in Kidney Transplant and Dialysis Patients: Results of the ERACODA Collaboration. Nephrol. Dial. Transplant..

[4] Badrouchi S., Barbouch S., Bettaieb A., Sellami N., Hajji M., Ben Abdallah T., Ben Hamida F., Harzallah A., Abderrahim E. (2022). Peritoneal Dialysis in the Era of COVID-19: Experience of a Tunisian Center. J. Nephrol..

[5] Fernandez-Prado R., Gonzalez-Parra E., Ortiz A. (2020). Often Forgotten, Transport Modality to Dialysis May Be Life-Saving. Clin. Kidney J..

[6] Quiroga B., Soler M.J., Ortiz A., Martínez Vaquera S., Jarava Mantecón C.J., Useche G., Sánchez Márquez M.G., Carnerero M., Jaldo Rodríguez M.T., Muñoz Ramos P. (2021). Safety and Immediate Humoral Response of COVID-19 Vaccines in Chronic Kidney Disease Patients: The SENCOVAC Study. Nephrol. Dial. Transplant..

[7] Quiroga B., Ortiz A., Cabezas-Reina C.J., Ruiz Fuentes M.C., Jiménez V.L., Larrondo S.Z., Toapanta N., Gómez M.M., de Sequera P., Sánchez-Álvarez E. (2022). Evolving Spectrum but Persistent High Mortality of COVID-19 among Patients on Kidney Replacement Therapy in the Vaccine Era: The Spanish COVID-19 KRT Registry. Clin. Kidney J..

[8] Quiroga B., Soler M.J., Ortiz A., Bernat A., Muñoz Díaz A.B., Jarava Mantecón C.J., Gómez Pérez V.O., Calderón González C., Cervienka M., Mazuecos A. (2022). Loss of Humoral Response 3 Months after SARS-CoV-2 Vaccination in the CKD Spectrum: The Multicentric SENCOVAC Study. Nephrol. Dial. Transplant..

[9] Quiroga B., Soler M.J., Ortiz A., Orero E., Tejedor S., Mantecón C.J.J., Gómez Pérez V.O., Marín Franco A.J., Alfaro Sánchez C., Puerta Carretero M. (2022). Humoral Response to Third Dose of SARS-CoV-2 Vaccines in the CKD Spectrum. CJASN.

[10] Quiroga B., Soler M.J., Ortiz A., Jaravaca Mantecón C.J., Nava Pérez N., Serra Martín M., Sato Y., Marin Franco A.J., Pazmiño Zambrano D.F., Lucena Valverde R. (2022). Anti-Spike Antibodies 3 Months after SARS-CoV-2 MRNA Vaccine Booster Dose in Patients on Hemodialysis: The Prospective SENCOVAC Study. Clin. Kidney J..

[11] Glowinska I., Labij-Reduta B., Juzwiuk J., Lukaszewicz M., Pietruczuk A., Poplawska A., Daniluk-Jamro A., Kakareko K., Rydzewska-Rosolowska A., Naumnik B. (2022). Factors Influencing Longevity of Humoral Response to SARS-CoV-2 Vaccination in Patients with End Stage Kidney Disease Receiving Renal Replacement Therapy. JCM.

[12] Boongird S., Setthaudom C., Kitpermkiat R., Prasongtanakij S., Srisala S., Chuengsaman P., Nongnuch A., Assanatham M., Kiertiburanakul S., Malathum K. (2022). Durability of Humoral and Cellular Immunity after an Extended Primary Series with Heterologous Inactivated SARS-CoV-2 Prime-Boost and ChAdOx1 NCoV-19 in Dialysis Patients (ICON3). Vaccines.

[13] Stumpf J., Schwöbel J., Lindner T., Anders L., Siepmann T., Karger C., Hüther J., Martin H., Müller P., Faulhaber-Walter R. (2022). Risk of Strong Antibody Decline in Dialysis and Transplant Patients after SARS-CoV-2mRNA Vaccination: Six Months Data from the Observational Dia-Vacc Study. Lancet Reg. Health Eur..

[14] Beilhack G., Monteforte R., Frommlet F., Reindl-Schwaighofer R., Strassl R., Vychytil A. (2022). Humoral Response to MRNA-1273 SARS-CoV-2 Vaccine in Peritoneal Dialysis Patients: Is Boostering After Six Months Adequate?. Front. Med..

[15] Housset P., Kubab S., Pardon A., Vittoz N., Bozman D.-F., Hanafi L., Caudwell V., Faucon A.-L. (2022). Waning but Persistent Humoral Response 6 Months after the Third Dose of the MRNA BNT162b2 Vaccine in Hemodialysis and Peritoneal Dialysis Patients. J. Nephrol..

[16] Murt A., Dinc H.O., Altiparmak M.R., Yalin S.F., Yadigar S., Parmaksiz E., Kocazeybek B., Pekpak M., Ataman M.R. (2022). Waning of SARS-CoV-2 Vaccine-Induced Immune Response over 6 Months in Peritoneal Dialysis Patients and the Role of a Booster Dose in Maintaining Seropositivity. Nephron.

[17] Bensouna I., Caudwell V., Kubab S., Acquaviva S., Pardon A., Vittoz N., Bozman D.-F., Hanafi L., Faucon A.-L., Housset P. (2022). SARS-CoV-2 Antibody Response After a Third Dose of the BNT162b2 Vaccine in Patients Receiving Maintenance Hemodialysis or Peritoneal Dialysis. Am. J. Kidney Dis..

[18] Bates T.A., Leier H.C., Lyski Z.L., McBride S.K., Coulter F.J., Weinstein J.B., Goodman J.R., Lu Z., Siegel S.A.R., Sullivan P. (2021). Neutralization of SARS-CoV-2 Variants by Convalescent and BNT162b2 Vaccinated Serum. Nat. Commun..

[19] Cheng C.-C., Platen L., Christa C., Tellenbach M., Kappler V., Bester R., Liao B.-H., Holzmann-Littig C., Werz M., Schönhals E. (2022). Improved SARS-CoV-2 Neutralization of Delta and Omicron BA.1 Variants of Concern after Fourth Vaccination in Hemodialysis Patients. Vaccines.

[20] da Silva E.S., Kohnen M., Gilson G., Staub T., Arendt V., Hilger C., Servais J.-Y., Charpentier E., Domingues O., Snoeck C.J. (2022). Pre-Omicron Vaccine Breakthrough Infection Induces Superior Cross-Neutralization against SARS-CoV-2 Omicron BA.1 Compared to Infection Alone. Int. J. Mol. Sci..

[21] Herman-Edelstein M., Ben-Dor N., Agur T., Guetta T., Raiter A., Meisel E., Alkeesh W., Ori Y., Rozen-Zvi B., Zingerman B. (2022). BNT162b2 Booster Vaccination Induced Immunity against SARS-CoV-2 Variants among Hemodialysis Patients. Vaccines.

[22] Attias P., Azzaoui I., El Karoui K., de La Selle A., Sokal A., Chappert P., Grimbert P., Fernandez I., Bouvier M., Samson C. (2022). Immune Responses after a Third Dose of MRNA Vaccine Differ in Virus-Naive versus SARS-CoV-2-Recovered Dialysis Patients. Clin. J. Am. Soc. Nephrol..

[23] Quiroga B., Giorgi M., Barril G. (2021). During COVID-19 Stay at Home Even If You Are a Hemodialysis Patient. Ther. Apher. Dial..

[24] Einbinder Y., Perl J., Nacasch N., Bnaya A., Shavit L., Erez D., Shashar M., Halperin T., Grupper A., Benchetrit S. (2022). Humoral Response and SARS-CoV-2 Infection Risk Following the Third and Fourth Doses of the BNT162b2 Vaccine in Dialysis Patients. Am. J. Nephrol..

[25] Niedźwiedzka-Rystwej P., Majchrzak A., Aksak-Wąs B., Serwin K., Czajkowski Z., Grywalska E., Korona-Głowniak I., Roliński J., Parczewski M. (2022). Programmed Cell Death-1/Programmed Cell Death-1 Ligand as Prognostic Markers of Coronavirus Disease 2019 Severity. Cells.

[26] Yao Z.Q., Moorman J.P. (2013). Immune Exhaustion and Immune Senescence: Two Distinct Pathways for HBV Vaccine Failure during HCV and/or HIV Infection. Arch. Immunol. Ther. Exp..

[27] Ponce P., Peralta R., Felix C., Pinto C., Pinto B., Matos J.F. (2022). Vaccination against SARS-CoV-2 in Haemodialysis Patients: Spike’s Ab Response and the Influence of BMI and Age. Int. J. Environ. Res. Public Health.

