# Supplementary material for: Long-Term Dynamic Humoral Response to SARS-CoV-2 mRNA Vaccines in Patients on Peritoneal Dialysis
Source: Vaccines (Basel). 2022 Oct 18;10(10):1738. doi: 10.3390/vaccines10101738 (PMC9609237; doi:10.3390/vaccines10101738)
Supplement: Supplementary file 1 [file vaccines-10-01738-s001.zip › vaccines-1938866-supplementary.pdf]

Supplementary Materials

Supplementary Table S1. Baseline characteristics of the participants included in each humoral response assessment.

	Baseline (n=102)	28 days (n=143)	3 months (n=139)	6 months (n=158)	12 months (n=164)
Age (years) (mean±standard deviation)	54±13	61±13	61±13	61±13	61±13
Sex (male) (%)	72 (71)	99 (69)	94 (68)	111 (70)	113 (69)
Diabetic kidney disease (%)	23 (23)	26 (18)	26 (19)	30 (19)	31 (19)
CAPD (%)	55 (54)	77 (54)	71 (51)	82 (52)	85 (52)
RAAS inhibitors (%)	72 (71)	92 (64)	86 (62)	97 (61)	101 (62)
ESA (%)	63 (62)	93 (65)	91 (66)	102 (65)	107 (65)
Vaccine (1ST/2ND doses) (%)	100 (100)	100 (100)	100 (100)	100 (100)	100 (100)
- BNT162b2	23 (23)	33 (23)	34 (24)	37 (23)	41 (25)
- mRNA-1273	79 (77)	110 (77)	105 (76)	121 (77)	123 (75)
Vaccine (3rd dose) (%)				102 (65)	164 (100)
- BNT162b2	--	--	--	19 (19)	31 (19)
- mRNA-1273				83 (81)	133 (81)
Vaccine (4th dose) (%)					44 (31)
- BNT162b2	--	--	--	--	23 (30)
- mRNA-1273					31 (70)

Abbreviations: CAPD: continuous ambulatory peritoneal dialysis; RAAS: renin angiotensin aldosterone system; ESA: erythropoietic stimulant agents.

SENCOVAC collaborative network:

Hospital Universitario Infanta Leonor: María Teresa Jaldo Rodríguez, Rafael Lucena Valverde, Marta Puerta Carretero, Mayra Ortega Díaz, Patricia Muñoz Ramos, Daniel Gaitán Tocora, Esther Rodriguez Suárez, Alfredo José Sáenz Santolaya, Patricia Arribas Cobo, Tamar Talavan, Raquel Cerrajero Calero.

Hospital Fundación Jiménez Díaz: Carolina Gracia-Iguacel, Emilio González-Parra, Mónica Pereira, Catalina Martin-Cleary, Ana Ramos-Verde, Jinny Sanchez-Rodriguez

Hospital Universitario de la Princesa: Martín Giorgi, Carmen Sánchez, Yohana Gil Giraldo, Ana Sánchez Horrillo, Pablo Ruano Suárez, Antonio Fernández Perpén, Andrés Fernández Ramos, Laura Salanova Villanueva, Alejandra Cortiñas, Ana Sánchez Horrillo, Pablo A. Díez Arias, Alicia Cabrera Cárdenas, Antonio de Santos, Almudena Núñez, Guillermina Barril Cuadrado, Loreto Mariscal de Gante, Raquel Repollet, Concepción Redondo Polo.

Hospital Vall D'Hebrón: Francesc Moreso, María Antonieta Azancot, Natalia Ramos, Oriol Bestard   Nestor Toapanta, Ignacio Cidraque, Sheila Bermejo, Irene Agraz, Oreto Prat, Carlota Medina, Emma Pardo, Oreto Prat, Alejandro Saiz.

Hospital Son Espases: Maria Antònia Munar Vila

Hospital Da Mariña: Secundino Cigarrán Guldris, Nicolás Menéndez Granados, María Jesús Corton Cabo, Walter López Alarcón

Hospital Rey Juan Carlos: Soledad Pizarro Sánchez, Simona Alexandru, Laura García Puente Suarez, Saul Pampa Saico, Marisol Poma Tapia

Hospital Universitario de Villalba: Rocío Zamora, Rosa Sánchez Hernández, Laura Rodríguez Osorio

Clínica Universidad de Navarra: Nuria García-Fernández, Paloma Leticia Martin Moreno, Noelia Ania González, Ana Sabalza Ortiz, María Nieves Bastida Iñarrea.

Hospital Reina Sofía de Córdoba: Sagrario Soriano Cabrera, Raquel Ojeda López, Cayetana Moyano Peregrí, Maria Luisa Agüera Morales, Maria Dolores Navarro Cabello.

Hospital Universitario Puerta del Mar: Auxiliadora Mazuecos, Juan Manuel Cazorla López, Teresa García, Carlos Narváez, Cristhian Orellana

Hospital Universitario Puerto Real: María Gabriela Sánchez Márquez, Carolina Lancho Novillo, Pedro Luis Quirós Ganga, Fernando Vallejo Carrión, Antonio Luis García Herrera

Hospital QuirónSalud A Coruña: Natalia Blanco Castro

Hospital Universitario de Badajoz: Rosa María Ruiz-Calero Cendrero, María Victoria Martín Hidalgo-Barquero, Román Hernández Gallego, Álvaro Alvarez, Eva Vázquez Leo

Diaverum Andalucía: Carlos Jesús Jarava Mantecón, Virginia Olinda Gomes Pérez, José Luis Pizarro León, Manuel Antonio Martínez García, Benaldina García Jiménez, Virginia Olinda Gómez Pérez, Juan de Dios Ramiro Moya, Diana López Espinosa, Alejandro Jiménez Herrador, Manuel Navarro Zurita, Leonardo Díaz Álvarez, Álvaro González Martínez, Sandra Báez Arroyo, Raquel Reina Fernández, Marlyn Janella Suárez Vargas, Rocío Calurano Casero.

Diaverum Valencia: Antoni Bordils, José Lacueva Gustavo Useche, Amparo Bernat García, Ana Beatriz Muñoz Díaz, Carmen Santamaría de Miguel, Ángel Palacios, Brenda Henningsmeyer, Esther Orero Calve, José Lacueva Moya, Yurika Sato, Marta Serra Marín, Sandra Tejedor.

Diaverum Murcia-Alicante: Shaida Martínez Vaquera, Pablo Delgado Conde, Manuel Carnerero di Riso, Irene Torres, Georgina Alfaro, Olga Halauko, Fouad El Rifai, Ana Dolores Martínez.

Diaverum Baleares: Pablo Justo Ávila

Diaverum Galicia – Castilla y León: Christian Alfaro Sánchez, Antonio José Marin Franco

Diaverum Madrid: Marta Sanz Sainz

Hospital Universitario San Agustín: José María Baltar Martín, Laura del Río García, José Luis Pérez Canga, Paola Milena Villabón Ochoa

Hospital Infanta Cristina: Laura Muñiz Pacios, Lina León Machado.

Consorci Sanitari Alt Penedès: Augusto Quiroz Morales, Ignacio Manzur Cavalotti, Itziar Navarro Zorita, Sol Otero López, Sara Outon González, Carlos Soto Montañez

Hospital Universitario de la Paz: Auxiliadora Bajo Rubio, Gloria Del Peso Gilsanz, Marta Ossorio Gonzalez, Rafael Sánchez Villanueva, Maria Ovidia Lopez Oliva

Hospital Lucus Augusti: Jesús Calviño Varela, Alba García Enríquez, Carmen Cobelo Casas, Pablo Otero Alonso, Lourdes González Tabares

Complejo Hospitalario Universitario de Vigo: José María Lamas Barreiro, Luisa Palomares Solla, Arginino Gándara, Walfred Nájera de la Garza, Francisco Fernández Fleming, María Gloria Rodríguez Goyanes, Cintia Caramés Feijoo, María Mercedes Moreiras Plaza, Cristina Bayón Juan

Hospital Universitario de Guadalajara: Gabriel de Arriba de la Fuente, Katia Pérez del Valle.

Hospital General de Alicante: Francisco Javier Pérez Contreras, Noelia Balibrea Lara, Dioné González Ferri, Eduardo Muñoz de Bustillo Llorente

Hospital Cruz Roja de Gijón: Enriqueta González Rodríguez

Hospital Clínico San Carlos: Virginia Lopez De La Manzanara Perez, Marta Calvo Arevalo, Jose Antonio Herrero Calvo

Hospital Universitario Puerta de Hierro: María Rosario Llópez Carratalá, Leyre María Martin Rodríguez, Marisa Serrano Salazar, Begoña Bravo Prieto, José María Portolés Pérez.

Hospital San Pedro: Antonio Gil Paraíso, Emma Huarte, M. Lanau

Fundación Hospital Calahorra: Rodrigo Avellaneda Campos, José Manuel Ubé

FMC San Rafael: Isabel Berdud Godoy, Esther Torres Aguilera, Rolando Tello Alea, Margie Soledad Del Rosario Saldaña.

Hospital Virgen Macarena de Sevilla: Mercedes Salgueira, Nuria Aresté, María de los Ángeles Rodríguez, Rocío Collantes, Ana Isabel Martínez, María Jesús Moyano, Elena Jiménez Víbora.

FMC Madrid-Dialcentro: Sandra Castellano Gash, Lara Ruíz Martínez

Hospital Universitario de Donosti: María Cinta Aguilar Cervera, María Teresa Rodrigo De Tomas, Beatriz Azcue Prieto, Carmen Toyos, José Molina Del Rio, Adriana Restrepo Acosta, Amagoia Celayeta Zamacona

Hospital de Galdakao: Mª Isabel Jimeno Martin, Saioa Bilbao Ortega, Mª Isabel Gallardo Ruiz, Ainhoa Hernando Rubio, Paula Garcia Ledesma, Alvaro Goyoaga Alvarez

Hospital Clinic de Barcelona: Esteban Poch, David Cucchiari, José Broseta Monzo.

Hospital Universitario de Canarias: Beatriz Escamilla Cabrera, Aurelio Pastor Rodríguez Hernández, María Sagrario García Rebollo, Juana Margarita Rufino Hernández

Hospital Lluis Alcanyis: Alejandra Yugueros González, Juan Carlos Alonso, Amparo Martínez Más, Manuel Calvé, Marina Garces Cardona, Vanesa Canto Balaguer

Hospital de Basurto: José Ignacio Minguela Pesquera

Hospital de Vinalopó: Eva Cotilla de la Rosa, David Rodríguez Santarelli, Alba Santos Garcia, Antonio Cabezas Martin-Caro, Inmaculada Martinez Santamaria

CAU Palencia: Michal Cervienka, Carmen Calderón González, Ana María Urraca de la Pisa, Lidia Sendino Monzon, Karina Ampuero Anachuri, Esther Hernández Garcia, Victoria Oviedo Gomez

Hospital Universitario Doctor Negrín: Raquel Santana Estupiñán, Tania Raquel Monzón Vázquez, Francisco Valga Amado, Patricia Pérez Borges

Hospital Arquitecto Marcide: Marta Durán Beloso, Fernanda Arroyo Alonso, Noemí Pérez Felpete, Ana Meizoso Ameneiro, Mónica Cunha Mera

Hospital Universitario del Sureste: Beatriz Gil Casares

Hospital de Cruces: Sofía Zarraga Larrondo, Naroa Maruki Kareaga, Ainhoa Inza San Salvador del Valle, Ana Rosa Muñoz García

Clínica Santa Isabel: Blanca Villacorta Linaza, Nuria del Toro Espinosa, Paola Estupiñan Perico, José Manuel Sánchez Oliva

Complejo Hospitalario de Navarra: Joaquín Manrique, Itziar Castaño, Carolina Purroi Nerea Gómez, Cristina Mansilla, Ane Utzurrum

Consorci Sanitari de Terrasa: Manuel Ramírez de Arellano Serna

Fundación Puigvert: Luis Guirado Perich

Hospital Clínico San Carlos: María Muñiz Rincón, Virginia Lopez De La Manzanara Perez, Marta Calvo Arevalo,
Jose Antonio Herrero Calvo

Complejo Hospitalario de Zamora: Jesús Grande Villoria

Hospital Álvarez Buylla de Mieres: Alfonso Pobes Martínez De Salinas
